# Associations between *Shokuiku* during School Years, Well-Balanced Diets, and Eating and Lifestyle Behaviours in Japanese Females Enrolled in a University Registered Dietitian Course

**DOI:** 10.3390/nu16040484

**Published:** 2024-02-07

**Authors:** Etsuko Kibayashi, Makiko Nakade

**Affiliations:** 1 Department of Food and Nutrition, Sonoda Women’s University, Amagasaki 661-8520, Hyogo, Japan; 2 Department of Food Science and Nutrition, University of Hyogo, Himeji 670-0092, Hyogo, Japan; nakade@shse.u-hyogo.ac.jp; 3 Research Institute for Food and Nutritional Sciences, Himeji 670-0092, Hyogo, Japan

**Keywords:** female university students, well-balanced diets, *shokuiku* (nutritional education), living alone, lifestyle behaviours

## Abstract

This study comprehensively examined the associations between *shokuiku* (food and nutrition education) during school years, current well-balanced diets, and current eating and lifestyle behaviours of Japanese female university students. A hypothetical model was developed using factors potentially associated with well-balanced diets. A simultaneous multipopulational analysis was performed according to the living arrangements of 148 female Japanese students (48.6% living alone) from a registered dietitian course. The analysis showed acceptable goodness of fit and a significant positive path from *shokuiku* during school years (living alone: standardised estimate 0.29, *p* = 0.004; with family: 0.32, *p* = 0.006) and a negative path from eating out frequency (−0.19, *p* = 0.039; −0.24, *p* = 0.017) towards a well-balanced diet. A significant negative path was identified from late bedtimes (−0.45, *p* < 0.001) and home meal replacement use frequency (−0.24, *p* = 0.010) in those living alone and from late-night snacking frequency (−0.27, *p* = 0.007) in those living with family. Well-balanced diets in female university students may be positively associated with *shokuiku* during school years and limited by a late bedtime, eating out, and home meal replacement use in those living alone, and by late-night snacking and eating out in those living with family.

## 1. Introduction

With assistance from the Food and Agriculture Organization (FAO), more than 100 countries have developed food-based dietary guidelines to promote healthy dietary practices. These guidelines have been adapted to each country’s nutritional situation, food availability, culinary culture, and eating habits. Furthermore, several countries publish food guides, often in the form of food pyramids and food plates used in consumer education [1]. For example, in accordance with the revised 2010 Dietary Guidelines for Americans [2], “MyPlate” was developed in the United States with five food groups to form a food guide for selecting foods that comprise a healthy diet [3]. In a meta-analysis, diets that achieved high scores in adhering to the Dietary Guidelines for Americans [4] were associated with a significant reduction in the risk of all-cause mortality, cardiovascular disease, cancer, and type 2 diabetes [5].

In Japan, the food-based dietary guidelines for Japanese 2000 (partly revised in 2016) [6] recommended well-balanced meals composed of staples (cereal grains), main dishes (proteins), and sides (vegetables). In 2005, the “Dietary Balance Guide” was also formulated. The risk of death from cardiovascular disease [7,8] and cerebrovascular disease [8] is lower in individuals who adhere to the daily recommended amounts of various food groups by sex and age, as indicated in the 2005 Dietary Balance Guide [9]. In addition to the guidelines, the Basic Act on *Shokuiku* (food and nutrition education) was enacted in 2005 to promote *shokuiku* in Japan. In the same year, a “diet and nutrition teacher” system was established in elementary and junior high schools to enhance the *shokuiku* teaching programme. At elementary schools, the diet and nutrition teacher actively works to ensure a healthy diet based on *shokuiku* programmes. The focus is primarily on three items: (1) nutritional balance among the “three food groups” (staples, such as cereal grains and potatoes; proteins, such as meat, fish, eggs, soybeans/soybean products, and dairy products; and side dishes, such as fruits and vegetables); (2) the roles of major nutrients; and (3) the importance of breakfast. The Basic Act on *Shokuiku* states the importance of *shokuiku* at school, at home, in the community, etc.

The Japanese government has aimed to increase the proportion of Japanese individuals consuming meals comprising staple foods, main dishes, and side dishes at least twice daily to at least 50% of the population by 2025. However, in 2020, the actual figures remained extremely low at 36.4% [10]. The National Health and Nutrition Survey conducted in 2018 before the COVID-19 pandemic reported that the proportion of respondents aged 20 years or older who consumed meals composed of staples, main dishes, and sides at least twice daily “almost every day” was the lowest among males aged 30–39 years (34.7% vs. 40.9% of women aged 30–39), followed by males and females aged 20–29 years (38.6% and 38.9%, respectively), compared to men and females aged 60–69 years (49.2% and 51.2%, respectively); the proportion was the lowest among younger generations [11]. Therefore, there is a need to examine the factors hindering younger adults from eating well-balanced diets.

It has been reported that eating well-balanced meals composed of staples, main dishes, and sides at least twice daily is associated with a higher frequency of breakfast intake and a lower frequency of eating out among those aged 20–39 years [12]. Thus, regular breakfast consumption promotes the habit of eating a well-balanced diet; frequently eating out hinders this habit. Although one study [12] on participants aged 20–39 years found no association between the frequency of home meal replacement use (i.e., consumption of ready-to-eat foods) and the consumption of a well-balanced diet, the 2015 National Health and Nutrition Survey reported that those aged ≥20 years who regularly used home meal replacements and ate out were less frequent consumers of well-balanced meals [13]. Additionally, because of the COVID-19 pandemic, takeout and home-delivery food service sales have increased in Japan since 2020 [14].

Also, although studies on the Japanese population are lacking, a previous study suggested that Hispanic/Latino men eat lower-quality meals and more frequently eat meals not prepared at home than Hispanic/Latino women [15]. Moreover, Korean adolescents (aged 12–18 years) who eat much later at night show lower dietary quality [16], and healthy adolescents (aged 14–17 years) who have fewer sleeping hours consume more carbohydrates, added sugars, foods higher in glycaemic load, and sweet drinks, and eat fewer servings of fruits/vegetables [17].

Despite the active implementation of *shokuiku* during school years, the proportion of younger adults eating well-balanced diets is low; thus, there is a need to examine the associations between *shokuiku* during school years from 7 to 12 years old, their current eating and lifestyle behaviours, and their current diet to determine the reasons why few young adults have well-balanced diets. Furthermore, those living alone are likely to have dietary needs that differ from those not living alone [18]. Therefore, the consumption rates of well-balanced diets in young adults should be clarified in terms of differences between those living with family and those living alone.

University students are more independent from their parents and are more likely to experience changes in their lives. For example, some students move out of their parents’ house, start living alone, and have part-time jobs. Also, while parents often prepare meals for high school students, university students have more opportunities to choose what they eat on their own. According to a national survey, the largest percentage (25%) of Japanese people started skipping breakfast between the ages of 20 and 29, followed by after high school graduation (18.4%) [19]. A previous study has also reported a significant decrease in the number of university students who ate breakfast and those who ate well-balanced meals over a three-year period [20]. Therefore, among young adults, a study focusing on university students is important.

In this study, we examined the structural associations between *shokuiku* during school years and the current eating and lifestyle behaviours that hinder well-balanced diets, considering different living arrangements (living alone or with family). In this study, we focused on bedtime, snacking, late-night snacking, eating out, and home meal replacement use as factors that inhibit having a well-balanced diet. We aimed to comprehensively determine the associations between *shokuiku* during school years, current well-balanced diets, and eating and lifestyle behaviours that negatively influence the well-balanced diets of Japanese female university students.

## 2. Materials and Methods

### 2.1. Study Design and Participants

A self-administered questionnaire survey was conducted from August to November 2020 among first- to fourth-year students (18–23 years old, n = 162, approximately 40 students belonged to each school year) of a registered dietitian course at a university in Hyogo Prefecture, Japan. Questionnaires were distributed during breaks before or after classes, and responses were obtained from those who agreed to participate in the study. In total, 161 students (5 males and 156 females) responded to the survey (99.4% response rate). Among them, 148 female university students (19.9 ± 1.3 years, 48.6% living alone) without missing values were included in this cross-sectional study. Female students in a registered dietitian course were targeted to minimise the differences in food and nutrition knowledge.

### 2.2. Measures

The questionnaire for this study included age, self-reported height and body weight, living arrangement (living alone or with family), financial well-being (Stable/Somewhat stable/Cannot say either/Not very stable/Not at all stable), regularity of exercise, that is, at least 2 days per week of exercise for at least 30 min continuously over 1 year (Yes/No), frequency of cooking for oneself per week (6 or 7 days/4 or 5 days/2 or 3 days/1 day or less), frequency of eating meals with family or friends per week (6 or 7 days/4 or 5 days/2 or 3 days/1 day or less), regularity of breakfast consumption per week (6 or 7 days/4 or 5 days/2 or 3 days/1 day or less), and frequency of having well-balanced meals composed of staple, main dish, and sides at least twice daily (6 or 7 days/4 or 5 days/2 or 3 days/1 day or less). BMI (body mass index) was calculated as body weight (kg)/height (m)^2^.

As factors associated with well-balanced diets, *shokuiku* during school years (7 to 12 years old) and current eating and lifestyle behaviours were also included in the questionnaire. The items of *shokuiku* during school years (past conversation) were as follows: (1) I had conversations about nutritional balance consisting of the “three food groups” during school years, (2) I had conversations about the roles of major nutrients during school years, and (3) I had conversations about the importance of breakfast during school years. Respondents were asked to choose one of the options (Agree/Somewhat agree/Cannot say either/Not very agree/Not at all agree) for each item.

Current eating and lifestyle behaviour items were bedtime (the respondents filled in the time), snacking frequency (6 or 7 days/4 or 5 days/2 or 3 days/1 day or less), late-night snacking frequency (6 or 7 days/4 or 5 days/2 or 3 days/1 day), eating out frequency (6 or 7 days/4 or 5 days/2 or 3 days/1 day or less), and frequency of home meal replacement consumption [use of ready-to-eat foods] (6 or 7 days/4 or 5 days/2 or 3 days/1 day or less).

### 2.3. Data Analysis

Medians for the ordinal variable of age by living arrangement (living alone or with family) were compared using a Mann–Whitney *U*-test as it was determined not to be normally distributed by the Kolmogorov–Smirnov’s test (*p* < 0.05), and proportions (BMI category, financial well-being, and regularity of exercise) by living arrangement were compared using a chi-square test. The variables of frequency of cooking for oneself, frequency of eating meals with family or friends, regularity of breakfast consumption, having well-balanced diets, *shokuiku* during school years (with three variables), and current eating and lifestyle behaviours as limiting factors were analysed on an interval scale; Welch’s *t*-tests were therefore used to compare them by living arrangement.

**Figure 1:**
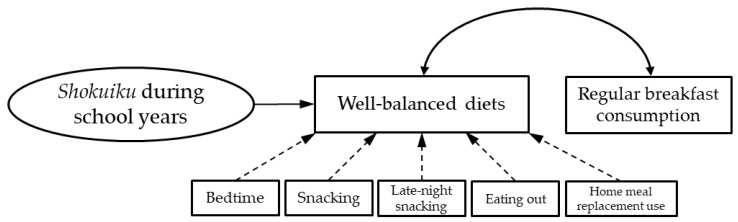
Initial hypothetical model of factors associated with well-balanced dietary habits. The bidirectional arc arrows indicate associations, the solid arrows indicate positive paths, and the dashed arrows indicate negative paths.

A hypothetical model based on living arrangements was developed for the association between *shokuiku* during school years and bedtime [17], snacking, late-night snacking [16], eating out [12,13], and home meal replacement use [13,15], which were inferred to hinder the current habit of having a well-balanced diet and the frequency of regular breakfast consumption. Accordingly, an initial hypothetical model was constructed, incorporating factors potentially associated with well-balanced diets, including *shokuiku* during school years (with three variables) and current eating and lifestyle behaviours as limiting factors (Figure 1). We added regular breakfast consumption to the hypothetical model because the importance of the meal is actively promoted through *shokuiku* during school years, and well-balanced meals are associated with a higher frequency of breakfast intake [12]. To validate the hypothetical model, we performed a covariance structure analysis as part of the overall and structural examinations of living arrangements (living alone or with family). We then conducted a simultaneous multipopulational analysis by living arrangement to check the goodness of fit of the model in each population and examined the configural invariance to confirm that the structure of the model was the same across the groups [21]. Differences in estimates by living arrangement were investigated through a pairwise comparison of parameters. Based on the path direction, standardised estimates, coefficient of determination, and fit indices such as the goodness of fit index (GFI), adjusted GFI (AGFI), comparative fit index (CFI), root mean square error of approximation (RMSEA), and Akaike’s information criterion (AIC), we repeatedly modified the model (such as by deleting nonsignificant paths) until the best possible fit was achieved. We judged the model’s goodness of fit to be better when the GFI, AGFI, and CFI indexes were ≥0.9, the RMSEA was ≤0.05, and the AIC was lower relative to those of multiple other models. The sample size was calculated using RMSEA for the null hypothesis: ε0 ≤ 0.1, and RMSEA for the alternative hypothesis: ε1 = 0.01, with power of the not close fit test = 0.8, model degrees of freedom = 40, a significance level of 5%. With 40 model degrees of freedom, the minimum sample size calculated was 74 [22]. Statistical significance was considered at the 5% level when the test statistic for the difference between parameters was ≥1.96. Statistical significance was set at *p* < 0.05. Statistical analyses were performed using SPSS (version 26, IBM Japan, Ltd., Tokyo, Japan, 2019).

## 3. Results

Table 1 shows the characteristics of the participants and the comparisons by living arrangement. Among participants who lived alone, those who cooked their meals 6 or 7 days/week accounted for the highest percentage (36.1%), whereas 69.7% of participants living with family cooked their meals no more than 1 day/week; there was a significant difference between the two groups (*p* < 0.001). In terms of eating with family or friends, 50.0% of participants living alone did so no more than 1 day/week, whereas 65.8% of participants living with family did so 6 or 7 days/week; there was a significant difference between the two groups (*p* < 0.001). A significantly higher percentage of those who lived alone (11.1%) than those who lived with family (1.3%) ate breakfast only 1 day/week or less (*p* < 0.001). The frequency of consumption of well-balanced meals composed of staples, main dishes, and sides at least twice daily, no more than 1 day/week, was the highest among participants living alone (45.8%) but the lowest among those living with family (18.4%); there was a significant difference between the two groups (*p* < 0.001).

Table 2 shows the results of comparisons of the factors potentially associated with well-balanced diets (*shokuiku* during school years, with current eating and lifestyle behaviours as the limiting factors) by living arrangement. A significant difference between those who lived alone and those living with family was observed only in bedtimes. Among participants living alone, those who went to bed after 2:00 am accounted for the highest percentage (44.4%), whereas among participants living with family, those who went to bed after 2:00 am accounted for a relatively low percentage (22.4%) (*p* = 0.001).

**Figure 2:**
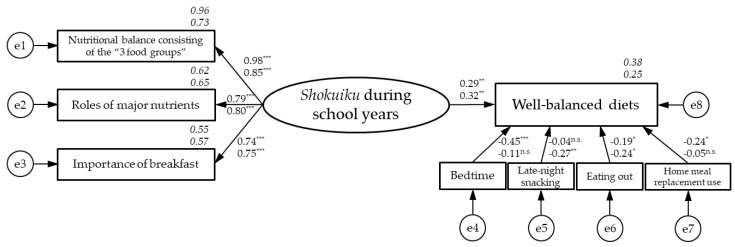
Associations between *shokuiku* during school years, current well-balanced diets, and current eating and lifestyle behaviours based on living arrangements (n = 148). Roman numbers in the path diagram indicate standardised estimates (next to the straight arrows). The numbers in italics are the *R^2^* values (coefficients of determination). Statistical significance was set at * *p* < 0.05, ** *p* < 0.01, and *** *p* < 0.001 (n.s., not significant). The upper number in each pair is the value for living alone (n = 72), and the bottom number of the pair is the value for living with family (n = 76). The results of simultaneous multipopulational analysis on two groups by living arrangement, living alone and with family, suggested that the hypothetical model had acceptable goodness of fit [χ^2^ = 49.168, *df* = 40 (*p* = 0.152), GFI = 0.918, AGFI = 0.852, CFI = 0.966, RMSEA = 0.040, AIC = 113.168].

We plotted the associations of *shokuiku* during school years, current well-balanced diets, and current eating and lifestyle behaviours with living arrangements (Figure 2). An analysis of the initial hypothetical model (Figure 1) for the overall combined residential status (living alone or with family) showed no acceptable goodness of fit for the model fit indices (GFI = 0.921, AGFI = 0.858, CFI = 0.899, RMSEA = 0.099, AIC = 80.966). Therefore, we deleted regular breakfast consumption (r = 0.103, *p* = 0.22) and snacking, which were not significantly associated with balanced diets. As a result, a simultaneous multipopulational analysis to examine the configural invariance by living arrangement showed acceptable goodness of fit (GFI = 0.918, AGFI = 0.852, CFI = 0.966, RMSEA = 0.040, AIC = 113.168), a significant positive path from *shokuiku* during school years (living alone: standardised estimate 0.29, *p* = 0.004; with family: 0.32, *p* = 0.006), and a negative path from eating out frequency (−0.19, *p* = 0.039; −0.24, *p* = 0.017) toward well-balanced diets (Figure 2). A significant negative path toward well-balanced diets was identified from bedtime (−0.45, *p* < 0.001) and home meal replacement consumption frequency (−0.24, *p* = 0.010) in those living alone and from late-night snacking frequency (−0.27, *p* = 0.007) in those living with family (Figure 2). The estimates of each path from bedtime, late-night snacking frequency, and home meal replacement use frequency toward well-balanced diets significantly differed by living arrangement.

## 4. Discussion

In this study, we examined the structural associations between *shokuiku* during school years, current well-balanced diets, and the current eating and lifestyle behaviours that hinder well-balanced diets in young adults. The results of our simultaneous multipopulational analysis focusing on the living arrangements of female students enrolled in a registered dietitian course suggested that while *shokuiku* during school years is correlated with current well-balanced eating habits, certain factors such as eating out (in both living arrangements), a late bedtime and home meal replacement use (when living alone), and late-night snacking (when living with family) may have negative associations.

We focused on the following three items in *shokuiku* during school years: (1) nutritional balance among the “three food groups” (staples, such as cereal grains and potatoes; proteins, such as meat, fish, eggs, soybeans/soybean products, and dairy products; and side dishes, such as fruits and vegetables); (2) the roles of the major nutrients; and (3) the importance of breakfast. A previous study [23] reported a self-efficacy scale for improving eating habits consisting of 12 items: “eating breakfast” had the highest percentage (93.4%) among second-year high school students, followed by “eating three meals a day” (88.4%), and “eating fruits and vegetables” (71.8%). The same study [23] reported a significant positive path from dietary and lifestyle habit self-efficacy to dietary habits via a stage of eating behaviour change. Dietary and lifestyle habit self-efficacy is a predisposing factor for behaviour and lifestyle [24]. Therefore, although regular breakfast consumption was not significantly associated with a current well-balanced diet, it is possible that *shokuiku* during school years has enhanced students’ self-efficacy in eating healthy foods, such as fruit and vegetables, and has contributed to their current adoption of well-balanced diets. Additionally, increased perceived competence in healthy eating and behavioural automaticity to consume healthy foods [25] may be acquired through *shokuiku* programmes at school and discussions on the knowledge acquired through these *shokuiku* programmes.

Our finding that eating out and home meal replacement use in those who lived alone had a negative association with well-balanced diets supports the results of a previous study [13], despite our study population having a high level of food awareness and food and nutrition knowledge. A previous study on factors associated with reduced food access reported that young individuals and households with single adults were significantly more likely to lack money for food [26]. Therefore, one reason why eating out and home meal replacement use hinder well-balanced diet adoption in those who live alone may be that access to such diets is financially limited because nutritious foods are generally perceived as more expensive [27].

Our findings that a late bedtime when living alone and late-night snacking when living with family hinder well-balanced diets may be related to adolescent and young adult chronotypes. The incidence of delayed bedtime peaks at around the ages of 17 to 20 years [28,29]. Among students living alone, a previous study reported an association between short sleep duration and infrequent breakfast intake [30]. Among students living with family, adolescents who ate much later at night (night eaters) showed lower Dietary Diversity Scores and were more likely to skip breakfast [16]. Well-balanced meals were associated with a higher frequency of breakfast intake [12]. Thus, skipping breakfast caused by a lack of sleep or night eating may negatively affect a well-balanced diet. In this study, regular breakfast consumption was not related to well-balanced diets. In Japan, well-balanced meals are defined as eating at least twice daily, so even if breakfast is skipped, eating well-balanced meals for lunch and dinner would be considered eating twice, and this may have affected the results of the association between breakfast and well-balanced diets.

The strength of our study was its structural examination of the associations of current eating and lifestyle behaviours that prevented the adoption of a well-balanced diet (at least by the female participants studied herein) and whether or not the respondents lived with family, in light of the associations of prerequisite factors (*shokuiku* during school years). However, our study had the following limitations: First, the participants were female students enrolled in a registered dietitian course at a university in Hyogo Prefecture—a group that had high levels of food and nutrition awareness/knowledge; therefore, this was not representative of the overall Japanese population aged 18–23 years. Second, because this was a cross-sectional study, we were unable to obtain a causal relationship between *shokuiku* during school years and later habituation to a well-balanced diet. In the future, our results should be confirmed in males and participants of differing ages.

## 5. Conclusions

Our results suggest that despite the positive impact of *shokuiku* during school years on the later adoption of well-balanced eating habits, even female registered dietitian course students who possess extensive knowledge regarding food and nutrition may be negatively affected by nocturnal lifestyles and eating out, as well as by home meal replacement use if they live alone. Future improvements in the dietary habits of young Japanese adults living alone may require that they adopt an earlier bedtime. Additional research is needed on the development of attractive food environments for consumers that facilitate access to healthy diets and well-balanced meals composed of staples, main dishes, and sides in the food service industry, including home meal replacements.

## Figures and Tables

**Table 1:** Characteristics of participants and comparison based on living arrangements (n = 148).

Characteristics	Total		Living Alone		Living with Family		*p*
	n = 148		n = 72		n = 76		
	n	%	n	%	n	%	
Age ^†^							
	20		20		20		0.27
	(19, 21)		(19, 21)		(19, 21)		
Body mass index (BMI, kg/m^2^) ^‡^							
<18.5	26	17.6	9	12.5	17	22.4	0.12
≥18.5 and <25	122	82.4	63	87.5	59	77.6	
≥25	0	0.0	0	0.0	0	0.0	
Financial well-being ^‡^							
Stable	25	16.9	11	15.3	14	18.4	0.84
Somewhat stable	72	48.6	35	48.6	37	48.7	
Cannot say either	30	20.3	16	22.2	14	18.4	
Not very stable	20	13.5	10	13.9	10	13.2	
Not at all stable	1	0.7	0	0.0	1	1.3	
Regular exercise ^‡^							
At least 2 days/week of exercise for at least 30 min continuously over 1 year							
Yes	20	13.5	11	15.3	9	11.8	0.54
No	128	86.5	61	84.7	67	88.2	
Cooking for oneself ^§^							
6 or 7 days/week	28	18.9	26	36.1	2	2.6	<0.001
4 or 5 days/week	25	16.9	21	29.2	4	5.3	
2 or 3 days/week	35	23.6	18	25.0	17	22.4	
1 day/week or less	60	40.5	7	9.7	53	69.7	
Eating meals with family or friends ^§^							
6 or 7 days/week	52	35.1	2	2.8	50	65.8	<0.001
4 or 5 days/week	24	16.2	7	9.7	17	22.4	
2 or 3 days/week	35	23.6	27	37.5	8	10.5	
1 day/week or less	37	25.0	36	50.0	1	1.3	
Regular breakfast consumption ^§^							
6 or 7 days/week	92	62.2	34	47.2	58	76.3	<0.001
4 or 5 days/week	27	18.2	18	25.0	9	11.8	
2 or 3 days/week	20	13.5	12	16.7	8	10.5	
1 day/week or less	9	6.1	8	11.1	1	1.3	
Well-balanced diets ^§^							
6 or 7 days/week	29	19.6	9	12.5	20	26.3	<0.001
4 or 5 days/week	33	22.3	12	16.7	21	27.6	
2 or 3 days/week	39	26.4	18	25.0	21	27.6	
1 day/week or less	47	31.8	33	45.8	14	18.4	

^†^ Values for age groups are medians (25th percentile, 75th percentile) based on a comparison of two independent samples using the Mann–Whitney U-test. ^‡^ Ratios were based on two independent-sample chi-square tests. ^§^ A Welch’s *t*-test was used, as the data were analysed using interval scales (4 = 6 or 7 days/week; 3 = 4 or 5 days/week; 2 = 2 or 3 days/week; 1 = 1 day/week or less).

**Table 2:** Comparison of factors potentially associated with well-balanced diets (*Shokuiku* during school years, with current eating and lifestyle behaviours as limiting factors), according to living arrangements (n = 148).

	Living Alone		Living with Family		*p*
	n = 72		n = 76		
	n	%	n	%	
*Shokuiku* during school years, i.e., past conversations about the following three items:					
1. Nutritional balance consisting of the “three food groups”					
Agree	22	30.6	17	22.4	0.58
Somewhat agree	14	19.4	22	28.9	
Cannot say either	17	23.6	10	13.2	
Not very agree	13	18.1	24	31.6	
Not at all agree	6	8.3	3	3.9	
2. Role of major nutrients					
Agree	15	20.8	13	17.1	0.93
Somewhat agree	22	30.6	28	36.8	
Cannot say either	12	16.7	11	14.5	
Not very agree	18	25.0	18	23.7	
Not at all agree	5	6.9	6	7.9	
3. Importance of breakfast					
Agree	32	44.4	20	26.3	0.24
Somewhat agree	14	19.4	29	38.2	
Cannot say either	13	18.1	8	10.5	
Not very agree	9	12.5	15	19.7	
Not at all agree	4	5.6	4	5.3	
Current eating and lifestyle behaviours as limiting factors					
Bedtime					
After 2:00 a.m.	32	44.4	17	22.4	0.001
From 1:00 a.m. to 2:00 a.m.	27	37.5	29	38.2	
From 0:00 a.m. to 1:00 a.m.	10	13.9	20	26.3	
Before 0:00 a.m.	3	4.2	10	13.2	
Snacking frequency (except for late-night snacking)					
6 or 7 days/week	13	18.1	24	31.6	0.10
4 or 5 days/week	20	27.8	17	22.4	
2 or 3 days/week	29	40.3	28	36.8	
1 day/week or less	10	13.9	7	9.2	
Late-night snacking frequency (from after dinner until bedtime)					
6 or 7 days/week	2	2.8	4	5.3	0.67
4 or 5 days/week	4	5.6	6	7.9	
2 or 3 days/week	18	25.0	14	18.4	
1 day/week or less	48	66.7	52	68.4	
Eating-out frequency					
6 or 7 days/week	0	0.0	0	0.0	0.053
4 or 5 days/week	2	2.8	0	0.0	
2 or 3 days/week	20	27.8	14	18.4	
1 day/week or less	50	69.4	62	81.6	
Home meal replacement (ready-to-eat foods) use frequency					
6 or 7 days/week	1	1.4	2	2.6	0.91
4 or 5 days/week	4	5.6	6	7.9	
2 or 3 days/week	25	34.7	19	25.0	
1 day/week or less	42	58.3	49	64.5	

A Welch’s *t*-test was used, as the data on the factors were analysed as interval scales in the covariance structure analysis. *Shokuiku* during school years interval scale: 5 = Agree; 4 = Somewhat agree; 3 = Cannot say either; 2 = Not very agree; 1 = Not at all agree. Bedtime interval scale: 4 = After 2:00 a.m.; 3 = From 1:00 a.m. to 2:00 a.m.; 2 = From 0:00 a.m. to 1:00 a.m.; 1 = Before 0:00 a.m. Other interval scales: 4 = 6 or 7 days/week; 3 = 4 or 5 days/week; 2 = 2 or 3 days/week; 1 = 1 day/week or less.

## Data Availability

The data in this study are available on request from the corresponding authors. The data are not publicly available due to confidentiality reasons.
